# *catena*-Poly[[bis­(2,2′-bipyridine-κ^2^ *N*,*N*′)cadmium(II)]-μ-9,10-dioxo­anthracene-1,5-disulfonato-κ^2^ *O* ^1^:*O* ^5^]

**DOI:** 10.1107/S1600536809046881

**Published:** 2009-11-14

**Authors:** Jia Jia, Youdi Zhang, Yanhui Zhao

**Affiliations:** a Department of Chemistry, Baicheng Normal College, Baicheng, Jilin 137000, People’s Republic of China; b College of Chemistry, Northeast Normal University, Changchun, Jilin 130024, People’s Republic of China; c College of Chemistry, Jilin Normal University, Siping, Jilin 136000, People’s Republic of China

## Abstract

The title complex, [Cd(C_14_H_6_O_8_S_2_)(C_10_H_8_N_2_)_2_]_*n*_, exhibits a chain-like polymeric structure with 9,10-dioxoanthracene-1,5-disulfonate anions bridging Cd^II^ atoms in a bis-monodentate mode. The Cd^II^ atom shows a distorted octa­hedral environment, with four N atoms from two chelating 2,2′-bipyridine ligands forming the equatorial plane and two sulfonate O atoms from two 9,10-dioxoanthracene-1,5-disulfonate anions occupying the apical positions. Weak C—H⋯O hydrogen-bonding contacts and π–π inter­actions [centroid–centroid distances = 3.6920 (12) and 3.7095 (12) Å] connect the complex mol­ecules into a three-dimensional supra­molecular framework.

## Related literature

For applications of organosulfonate-based metal complexes, see: Vaira *et al.* (2003 ▶). For a review on structural chemistry and properties of metal arenesulfonates, see: Cai (2004 ▶). For self-assembled structural motifs in coordination chemistry, see: Cai *et al.* (2001 ▶); Sun & Lees (2001 ▶); Swiegers & Malefetse (2000 ▶). For the synthetic procedure, see: Zhao *et al.* (2007 ▶).
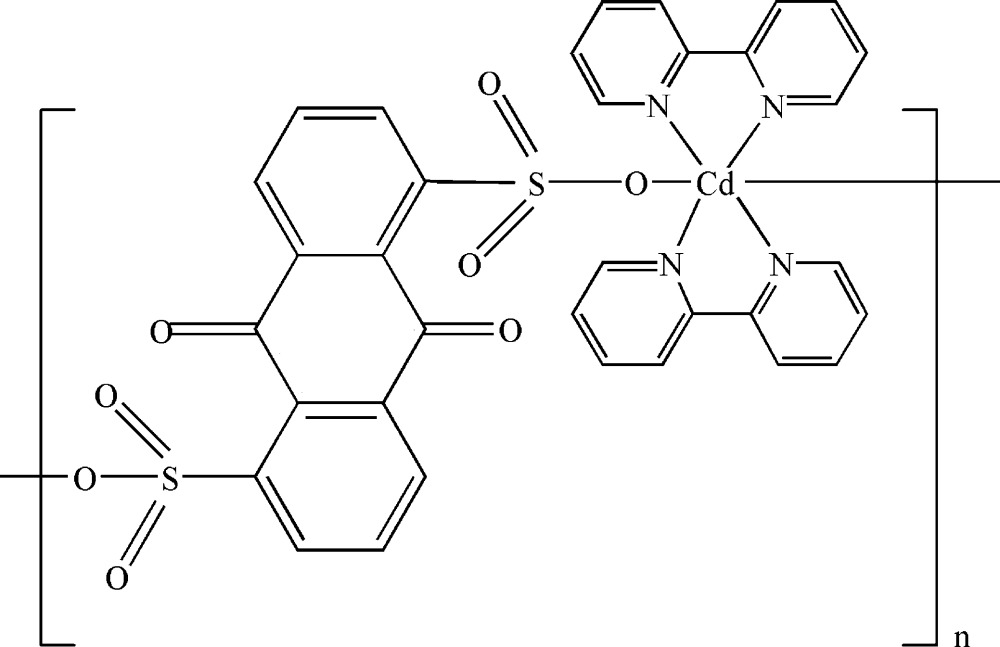


## Experimental

### 

#### Crystal data

[Cd(C_14_H_6_O_8_S_2_)(C_10_H_8_N_2_)_2_]
*M* *_r_* = 791.08
Triclinic, 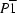

*a* = 10.3807 (7) Å
*b* = 10.7406 (8) Å
*c* = 13.1289 (9) Å
α = 94.044 (1)°
β = 90.239 (1)°
γ = 97.025 (1)°
*V* = 1449.08 (18) Å^3^
*Z* = 2
Mo *K*α radiation
μ = 0.96 mm^−1^
*T* = 296 K
0.24 × 0.23 × 0.22 mm

#### Data collection

Bruker APEXII CCD area-detector diffractometer
Absorption correction: multi-scan (*SADABS*; Sheldrick, 1996 ▶) *T*_min_ = 0.802, *T*_max_ = 0.816
7435 measured reflections
5078 independent reflections
4773 reflections with *I* > 2σ(*I*)
*R*_int_ = 0.010

#### Refinement

*R*[*F* ^2^ > 2σ(*F* ^2^)] = 0.022
*wR*(*F* ^2^) = 0.061
*S* = 1.01
5078 reflections
442 parameters
H-atom parameters constrained
Δρ_max_ = 0.29 e Å^−3^
Δρ_min_ = −0.47 e Å^−3^

### 

Data collection: *APEX2* (Bruker, 2003 ▶); cell refinement: *SAINT* (Bruker, 2001 ▶); data reduction: *SAINT*; program(s) used to solve structure: *SHELXS97* (Sheldrick, 2008 ▶); program(s) used to refine structure: *SHELXL97* (Sheldrick, 2008 ▶) and *PLATON* (Spek, 2009 ▶); molecular graphics: *SHELXTL* (Sheldrick, 2008 ▶) and *DIAMOND* (Brandenburg & Berndt, 1999 ▶); software used to prepare material for publication: *SHELXL97*.

## Supplementary Material

Crystal structure: contains datablocks I, global. DOI: 10.1107/S1600536809046881/si2219sup1.cif

Structure factors: contains datablocks I. DOI: 10.1107/S1600536809046881/si2219Isup2.hkl

Additional supplementary materials:  crystallographic information; 3D view; checkCIF report

## Figures and Tables

**Table 1:** Selected bond lengths (Å)

Cd1—N3	2.2645 (17)
Cd1—N1	2.3050 (17)
Cd1—N2	2.3356 (17)
Cd1—O4^i^	2.3527 (16)
Cd1—N4	2.3882 (17)
Cd1—O2	2.4109 (16)

Symmetry code: (i) 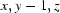.

**Table 2:** Hydrogen-bond geometry (Å, °)

*D*—H⋯*A*	*D*—H	H⋯*A*	*D*⋯*A*	*D*—H⋯*A*
C4—H4⋯O5^ii^	0.93	2.48	3.200 (3)	135
C5—H5⋯O4^ii^	0.93	2.53	3.239 (3)	133
C14—H14⋯O2^iii^	0.93	2.54	3.267 (3)	136

Symmetry codes: (ii) 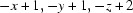; (iii) 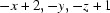.

**Fig. 1.**
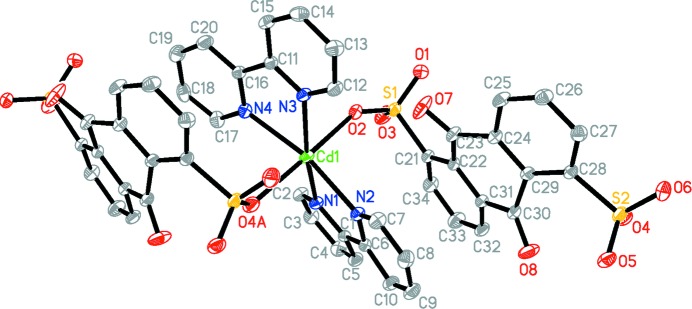
The local coordination environment of CdII in (I) drawn with 30% probability displacement ellipsoids. H atoms were omitted for clarity.

**Fig. 2.**
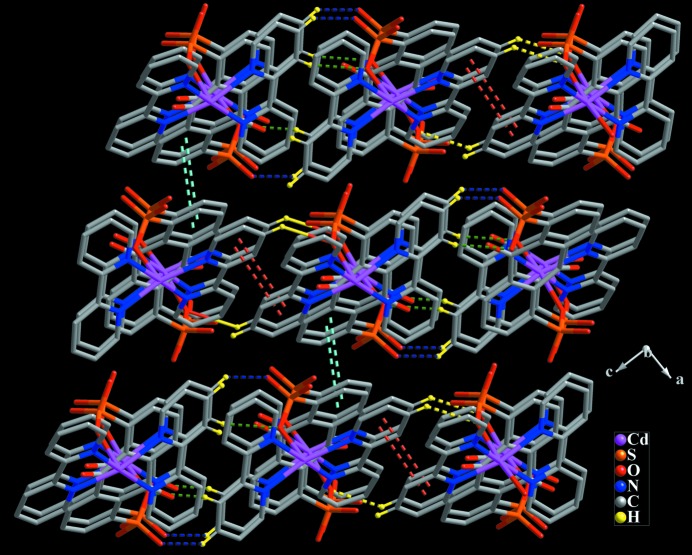
The three-dimensional supramolecular network of (I) produced by hydrogen-bonding and π–π stacking interactions.

## supplementary crystallographic information

### Comment

The chemistry of metal sulfonate compounds has been a research field of rapid expansion in recent years, mainly due to their adjustable coordination ability and interesting applications as functional materials [Cai, 2004; Vaira *et al.*, 2003; Zhao *et al.*, 2007]. Coordination interaction, H-bonding and π–π interaction usually play important roles in constructing, tuning, modifying, controlling, and modulating such coordination architectures [Cai *et al.*, 2001; Sun & Lees, 2001; Swiegers & Malefetse, 2000]. We herein report the crystal structure of a Cd ^II^ complex with 2,2'-bipyridine and 9,10-dioxoanthracene-1,5-disulfonate ligands (I).

The local coordination environment of Cd^II^ atom in I is shown in Fig.1. The unique Cd^II^ atom is six-coordinated by four N atoms from two unsymmetric bi-chelating 2,2'-bipyridine ligands and two sulfonate O atoms from two independent anthraquinone-1,5-disulfonate anions, exhibiting a slightly distorted octahedral coordination mode (Table 1). The anthraquinone-1,5-disulfonate anion adopts a bis-monodentate mode, linking the adjacent Cd ^II^ atoms into a one-dimentional infinite chain along the *b*-axis (Fig.2). The π–π stacking interactions between the adjacent 2,2'-bipyridine rings as well as weak C—H···O hydrogen-bonding interactions (Table 2) were observed (Fig. 2), which further extend the chains into a two-dimensional plane. Cg5···Cg5^iii^ distance is 3.6920 (12) Å, the perpendicular distance between the inversion-related planes (therefore the dihedral angle is zero) is 3.291 Å, and the slippage (offset) is 1.674 Å. Cg5 is the centroid of the ring (N3, C11, C15, C14, C13, C12), and the symmetry code iii = 2 - *x*, -*y*, 1 - *z*. Additionally, the adjacent two-dimensional planes are extended into a three-dimensional supramolecular network by π–π stacking interactions between the anthraquinone rings. Cg9···Cg9^iv^ distance is 3.7095 (12) Å, the plane-to-plane (perpendicular) distance between inversion related planes is 3.340 Å, the slippage is 1.615 Å, Cg9 is the centroid of the ring (C24, C25, C26, C27, C28, C29) and the symmetry code iv = 1 - *x*, 1 - *y*, 1 - *z*.

### Experimental

A mixture of disodium 9,10-dioxoanthracene-1,5-disulfonate (41.2 mg, 0.1 mmol), Cd(OAc)_2_.2H_2_O(26.7 mg, 0.1 mmol), 2,2'-bipyridine(15.6 mg, 0.1 mmol), and H_2_O (10 ml) was sealed in a 23 ml teflonlined stainlesssteel vessel. The vessel was heated to 413 K for 2 days under autogenous pressure and then cooled to room temperature at a rate of 2.4 K / h. Pink block-shaped crystals suitable for X-ray analysis were obtained in a 39% yield. Analysis calculated for C_34_H_22_CdN_4_O_8_S_2_: C 51.62, H 2.80, N 7.08%; found: C 51.53, H 2.84, N 6.99%.

### Refinement

H atoms were located in difference maps, but were subsequently placed in calculated positions and treated as riding, with C–H = 0.93 Å and O–H = 0.85 Å. All H atoms were allocated displacement parameters related to those of their parent atoms [*U*_iso_(H) = *U*_eq_(C, O)].

### Crystal data

**Table:**

[Cd(C_14_H_6_O_8_S_2_)(C_10_H_8_N_2_)_2_]	*Z* = 2
*M**_r_* = 791.08	*F*(000) = 796
Triclinic, *P*1	*D*_x_ = 1.813 Mg m^−^^3^
Hall symbol: -P 1	Mo *K*α radiation, λ = 0.71073 Å
*a* = 10.3807 (7) Å	Cell parameters from 6497 reflections
*b* = 10.7406 (8) Å	θ = 2.5–27.9°
*c* = 13.1289 (9) Å	µ = 0.96 mm^−^^1^
α = 94.044 (1)°	*T* = 296 K
β = 90.239 (1)°	BLOCK, pink
γ = 97.025 (1)°	0.24 × 0.23 × 0.22 mm
*V* = 1449.08 (18) Å^3^	

### Data collection

**Table:**

Bruker APEXII CCD area-detector diffractometer	5078 independent reflections
Radiation source: fine-focus sealed tube	4773 reflections with *I* > 2σ(*I*)
graphite	*R*_int_ = 0.010
φ and ω scans	θ_max_ = 25.0°, θ_min_ = 1.6°
Absorption correction: multi-scan (*SADABS*; Sheldrick, 1996)	*h* = −12→10
*T*_min_ = 0.802, *T*_max_ = 0.816	*k* = −12→12
7435 measured reflections	*l* = −14→15

### Refinement

**Table:**

Refinement on *F*^2^	Primary atom site location: structure-invariant direct methods
Least-squares matrix: full	Secondary atom site location: difference Fourier map
*R*[*F*^2^ > 2σ(*F*^2^)] = 0.022	Hydrogen site location: inferred from neighbouring sites
*wR*(*F*^2^) = 0.061	H-atom parameters constrained
*S* = 1.01	*w* = 1/[σ^2^(*F*_o_^2^) + (0.0345*P*)^2^ + 0.8688*P*] where *P* = (*F*_o_^2^ + 2*F*_c_^2^)/3
5078 reflections	(Δ/σ)_max_ = 0.001
442 parameters	Δρ_max_ = 0.29 e Å^−^^3^
0 restraints	Δρ_min_ = −0.47 e Å^−^^3^

### Special details

**Table:**

Geometry. All e.s.d.'s (except the e.s.d. in the dihedral angle between two l.s. planes) are estimated using the full covariance matrix. The cell e.s.d.'s are taken into account individually in the estimation of e.s.d.'s in distances, angles and torsion angles; correlations between e.s.d.'s in cell parameters are only used when they are defined by crystal symmetry. An approximate (isotropic) treatment of cell e.s.d.'s is used for estimating e.s.d.'s involving l.s. planes.
Refinement. Refinement of *F*^2^ against ALL reflections. The weighted *R*-factor *wR* and goodness of fit *S* are based on *F*^2^, conventional *R*-factors *R* are based on *F*, with *F* set to zero for negative *F*^2^. The threshold expression of *F*^2^ > σ(*F*^2^) is used only for calculating *R*-factors(gt) *etc*. and is not relevant to the choice of reflections for refinement. *R*-factors based on *F*^2^ are statistically about twice as large as those based on *F*, and *R*- factors based on ALL data will be even larger.

### Fractional atomic coordinates and isotropic or equivalent isotropic displacement parameters (Å^2^)

**Table:**

	*x*	*y*	*z*	*U*_iso_*/*U*_eq_	
Cd1	0.739498 (14)	−0.019562 (14)	0.758892 (11)	0.02811 (7)	
S1	0.98757 (5)	0.25760 (5)	0.82102 (4)	0.03023 (12)	
S2	0.49276 (5)	0.76753 (5)	0.66792 (4)	0.03213 (13)	
O1	1.06004 (17)	0.33125 (16)	0.74831 (15)	0.0487 (4)	
O2	0.92846 (15)	0.13520 (14)	0.77782 (13)	0.0374 (4)	
O3	1.06023 (18)	0.24621 (18)	0.91345 (14)	0.0515 (5)	
O4	0.56626 (15)	0.81750 (15)	0.76110 (13)	0.0416 (4)	
O5	0.36219 (14)	0.71481 (15)	0.68755 (13)	0.0399 (4)	
O6	0.50253 (18)	0.85773 (17)	0.59052 (15)	0.0529 (5)	
O7	0.8128 (2)	0.29015 (19)	0.64176 (13)	0.0602 (6)	
O8	0.49447 (18)	0.5670 (2)	0.82193 (14)	0.0557 (5)	
N1	0.73658 (17)	0.02158 (16)	0.93351 (13)	0.0282 (4)	
N2	0.57917 (17)	0.11075 (16)	0.79681 (13)	0.0288 (4)	
N3	0.79152 (17)	−0.03642 (17)	0.59157 (13)	0.0287 (4)	
N4	0.87250 (17)	−0.18502 (17)	0.73225 (14)	0.0304 (4)	
C1	0.6531 (2)	0.09907 (19)	0.97055 (15)	0.0267 (4)	
C2	0.8247 (2)	−0.0124 (2)	0.99750 (17)	0.0348 (5)	
H2	0.8824	−0.0662	0.9718	0.042*	
C3	0.8343 (2)	0.0280 (2)	1.09904 (17)	0.0377 (5)	
H3	0.8970	0.0026	1.1410	0.045*	
C4	0.7489 (2)	0.1070 (2)	1.13697 (17)	0.0397 (5)	
H4	0.7529	0.1361	1.2054	0.048*	
C5	0.6570 (2)	0.1427 (2)	1.07233 (17)	0.0365 (5)	
H5	0.5981	0.1957	1.0971	0.044*	
C6	0.5603 (2)	0.14002 (19)	0.89629 (16)	0.0272 (4)	
C7	0.4978 (2)	0.1472 (2)	0.72820 (18)	0.0366 (5)	
H7	0.5097	0.1257	0.6593	0.044*	
C8	0.3976 (2)	0.2151 (2)	0.75536 (19)	0.0396 (5)	
H8	0.3445	0.2410	0.7058	0.048*	
C9	0.3776 (2)	0.2439 (2)	0.8570 (2)	0.0415 (6)	
H9	0.3095	0.2884	0.8775	0.050*	
C10	0.4594 (2)	0.2061 (2)	0.92850 (18)	0.0369 (5)	
H10	0.4471	0.2247	0.9978	0.044*	
C11	0.86840 (19)	−0.12295 (19)	0.55847 (16)	0.0272 (4)	
C12	0.7463 (2)	0.0352 (2)	0.52407 (17)	0.0343 (5)	
H12	0.6919	0.0936	0.5471	0.041*	
C13	0.7761 (2)	0.0268 (2)	0.42227 (17)	0.0386 (5)	
H13	0.7431	0.0785	0.3773	0.046*	
C14	0.8560 (2)	−0.0599 (2)	0.38872 (17)	0.0382 (5)	
H14	0.8791	−0.0669	0.3204	0.046*	
C15	0.9019 (2)	−0.1368 (2)	0.45681 (17)	0.0345 (5)	
H15	0.9547	−0.1970	0.4346	0.041*	
C16	0.9134 (2)	−0.2036 (2)	0.63649 (16)	0.0286 (4)	
C17	0.9074 (2)	−0.2606 (2)	0.80218 (18)	0.0378 (5)	
H17	0.8783	−0.2487	0.8686	0.045*	
C18	0.9840 (2)	−0.3544 (2)	0.7803 (2)	0.0414 (6)	
H18	1.0055	−0.4053	0.8307	0.050*	
C19	1.0281 (3)	−0.3716 (2)	0.6829 (2)	0.0479 (6)	
H19	1.0814	−0.4334	0.6662	0.058*	
C20	0.9924 (2)	−0.2958 (2)	0.6098 (2)	0.0453 (6)	
H20	1.0210	−0.3064	0.5431	0.054*	
C21	0.8573 (2)	0.34568 (19)	0.86396 (16)	0.0279 (4)	
C22	0.77142 (19)	0.39712 (18)	0.79997 (16)	0.0261 (4)	
C23	0.7662 (2)	0.3762 (2)	0.68705 (17)	0.0328 (5)	
C24	0.70328 (19)	0.46704 (19)	0.62757 (16)	0.0275 (4)	
C25	0.7262 (2)	0.4667 (2)	0.52374 (17)	0.0356 (5)	
H25	0.7732	0.4067	0.4923	0.043*	
C26	0.6798 (2)	0.5545 (2)	0.46681 (17)	0.0385 (5)	
H26	0.6954	0.5543	0.3971	0.046*	
C27	0.6094 (2)	0.6439 (2)	0.51434 (17)	0.0344 (5)	
H27	0.5816	0.7057	0.4764	0.041*	
C28	0.58020 (19)	0.64261 (19)	0.61615 (16)	0.0269 (4)	
C29	0.62537 (19)	0.55178 (19)	0.67490 (16)	0.0262 (4)	
C30	0.5920 (2)	0.5344 (2)	0.78358 (17)	0.0308 (5)	
C31	0.6830 (2)	0.47193 (19)	0.84556 (16)	0.0279 (4)	
C32	0.6774 (2)	0.4918 (2)	0.95107 (17)	0.0358 (5)	
H32	0.6160	0.5393	0.9801	0.043*	
C33	0.7620 (3)	0.4417 (2)	1.01259 (18)	0.0436 (6)	
H33	0.7591	0.4558	1.0832	0.052*	
C34	0.8518 (2)	0.3697 (2)	0.96818 (17)	0.0372 (5)	
H34	0.9099	0.3367	1.0099	0.045*	

### Atomic displacement parameters (Å^2^)

**Table:**

	*U*^11^	*U*^22^	*U*^33^	*U*^12^	*U*^13^	*U*^23^
Cd1	0.03081 (10)	0.03273 (10)	0.02167 (9)	0.00993 (6)	0.00363 (6)	−0.00262 (6)
S1	0.0275 (3)	0.0272 (3)	0.0373 (3)	0.0088 (2)	0.0022 (2)	0.0019 (2)
S2	0.0279 (3)	0.0281 (3)	0.0418 (3)	0.0109 (2)	0.0022 (2)	−0.0007 (2)
O1	0.0451 (10)	0.0372 (9)	0.0651 (12)	0.0067 (8)	0.0230 (9)	0.0077 (8)
O2	0.0375 (9)	0.0279 (8)	0.0473 (10)	0.0077 (7)	0.0068 (7)	−0.0008 (7)
O3	0.0477 (10)	0.0599 (12)	0.0507 (11)	0.0287 (9)	−0.0136 (8)	−0.0065 (9)
O4	0.0343 (9)	0.0393 (9)	0.0485 (10)	0.0036 (7)	0.0039 (7)	−0.0134 (8)
O5	0.0265 (8)	0.0432 (9)	0.0504 (10)	0.0110 (7)	0.0016 (7)	−0.0048 (8)
O6	0.0564 (11)	0.0424 (10)	0.0667 (12)	0.0250 (9)	0.0120 (9)	0.0186 (9)
O7	0.0969 (16)	0.0589 (12)	0.0339 (9)	0.0549 (12)	−0.0067 (10)	−0.0112 (9)
O8	0.0515 (11)	0.0800 (14)	0.0464 (11)	0.0417 (10)	0.0220 (9)	0.0206 (10)
N1	0.0290 (9)	0.0311 (9)	0.0250 (9)	0.0072 (7)	0.0027 (7)	0.0002 (7)
N2	0.0295 (9)	0.0299 (9)	0.0274 (9)	0.0069 (7)	0.0019 (7)	−0.0001 (7)
N3	0.0308 (9)	0.0317 (9)	0.0248 (9)	0.0101 (7)	0.0034 (7)	−0.0003 (7)
N4	0.0336 (10)	0.0285 (9)	0.0295 (10)	0.0059 (7)	−0.0007 (8)	0.0007 (7)
C1	0.0289 (10)	0.0265 (10)	0.0250 (10)	0.0037 (8)	0.0072 (8)	0.0024 (8)
C2	0.0358 (12)	0.0398 (13)	0.0305 (11)	0.0133 (10)	0.0011 (9)	0.0002 (10)
C3	0.0445 (13)	0.0423 (13)	0.0275 (11)	0.0094 (11)	−0.0034 (10)	0.0051 (10)
C4	0.0572 (15)	0.0431 (13)	0.0197 (11)	0.0107 (11)	0.0026 (10)	0.0005 (9)
C5	0.0453 (13)	0.0377 (12)	0.0281 (11)	0.0126 (10)	0.0082 (10)	0.0007 (10)
C6	0.0279 (10)	0.0231 (10)	0.0301 (11)	0.0026 (8)	0.0040 (9)	−0.0005 (8)
C7	0.0379 (12)	0.0421 (13)	0.0306 (12)	0.0098 (10)	−0.0025 (10)	0.0004 (10)
C8	0.0348 (12)	0.0384 (13)	0.0465 (14)	0.0091 (10)	−0.0090 (10)	0.0022 (11)
C9	0.0314 (12)	0.0394 (13)	0.0546 (15)	0.0138 (10)	0.0000 (11)	−0.0060 (11)
C10	0.0358 (12)	0.0382 (12)	0.0372 (13)	0.0099 (10)	0.0061 (10)	−0.0040 (10)
C11	0.0252 (10)	0.0275 (10)	0.0289 (11)	0.0039 (8)	0.0031 (8)	−0.0003 (9)
C12	0.0364 (12)	0.0382 (12)	0.0307 (12)	0.0141 (10)	0.0035 (9)	0.0033 (10)
C13	0.0379 (13)	0.0499 (14)	0.0307 (12)	0.0121 (11)	−0.0001 (10)	0.0091 (10)
C14	0.0409 (13)	0.0504 (14)	0.0233 (11)	0.0062 (11)	0.0050 (10)	0.0010 (10)
C15	0.0361 (12)	0.0370 (12)	0.0308 (12)	0.0093 (10)	0.0069 (9)	−0.0040 (10)
C16	0.0252 (10)	0.0284 (11)	0.0324 (11)	0.0040 (8)	0.0034 (9)	0.0008 (9)
C17	0.0479 (14)	0.0351 (12)	0.0296 (12)	0.0025 (10)	−0.0046 (10)	0.0028 (10)
C18	0.0426 (13)	0.0315 (12)	0.0507 (15)	0.0031 (10)	−0.0137 (11)	0.0100 (11)
C19	0.0462 (15)	0.0433 (14)	0.0590 (17)	0.0209 (12)	0.0053 (12)	0.0102 (12)
C20	0.0475 (14)	0.0459 (14)	0.0477 (15)	0.0226 (12)	0.0161 (12)	0.0090 (12)
C21	0.0288 (11)	0.0253 (10)	0.0302 (11)	0.0063 (8)	0.0022 (9)	0.0002 (8)
C22	0.0271 (10)	0.0222 (10)	0.0291 (11)	0.0047 (8)	0.0022 (8)	0.0001 (8)
C23	0.0364 (12)	0.0311 (11)	0.0323 (12)	0.0140 (9)	0.0003 (9)	−0.0055 (9)
C24	0.0261 (10)	0.0295 (11)	0.0271 (11)	0.0075 (8)	−0.0017 (8)	−0.0034 (8)
C25	0.0370 (12)	0.0433 (13)	0.0280 (11)	0.0167 (10)	0.0010 (9)	−0.0073 (10)
C26	0.0417 (13)	0.0506 (14)	0.0249 (11)	0.0139 (11)	0.0027 (10)	0.0010 (10)
C27	0.0332 (12)	0.0387 (12)	0.0333 (12)	0.0101 (10)	−0.0007 (9)	0.0057 (10)
C28	0.0222 (10)	0.0276 (10)	0.0310 (11)	0.0060 (8)	−0.0008 (8)	−0.0021 (9)
C29	0.0230 (10)	0.0268 (10)	0.0287 (11)	0.0047 (8)	−0.0002 (8)	−0.0025 (8)
C30	0.0327 (11)	0.0281 (11)	0.0330 (11)	0.0105 (9)	0.0057 (9)	0.0001 (9)
C31	0.0291 (11)	0.0255 (10)	0.0293 (11)	0.0053 (8)	0.0039 (9)	−0.0001 (8)
C32	0.0406 (13)	0.0355 (12)	0.0329 (12)	0.0138 (10)	0.0080 (10)	−0.0031 (10)
C33	0.0538 (15)	0.0522 (15)	0.0266 (12)	0.0178 (12)	0.0011 (11)	−0.0035 (11)
C34	0.0423 (13)	0.0407 (13)	0.0304 (12)	0.0130 (10)	−0.0032 (10)	0.0016 (10)

### Geometric parameters (Å, °)

**Table:**

Cd1—N3	2.2645 (17)	C10—H10	0.9300
Cd1—N1	2.3050 (17)	C11—C15	1.383 (3)
Cd1—N2	2.3356 (17)	C11—C16	1.496 (3)
Cd1—O4^i^	2.3527 (16)	C12—C13	1.373 (3)
Cd1—N4	2.3882 (17)	C12—H12	0.9300
Cd1—O2	2.4109 (16)	C13—C14	1.373 (3)
S1—O1	1.4387 (18)	C13—H13	0.9300
S1—O3	1.4465 (18)	C14—C15	1.380 (3)
S1—O2	1.4564 (16)	C14—H14	0.9300
S1—C21	1.813 (2)	C15—H15	0.9300
S2—O5	1.4353 (17)	C16—C20	1.389 (3)
S2—O6	1.4481 (19)	C17—C18	1.374 (3)
S2—O4	1.4719 (18)	C17—H17	0.9300
S2—C28	1.806 (2)	C18—C19	1.368 (4)
O4—Cd1^ii^	2.3527 (16)	C18—H18	0.9300
O7—C23	1.215 (3)	C19—C20	1.379 (4)
O8—C30	1.211 (3)	C19—H19	0.9300
N1—C2	1.340 (3)	C20—H20	0.9300
N1—C1	1.343 (3)	C21—C34	1.377 (3)
N2—C6	1.342 (3)	C21—C22	1.409 (3)
N2—C7	1.343 (3)	C22—C31	1.402 (3)
N3—C12	1.334 (3)	C22—C23	1.483 (3)
N3—C11	1.348 (3)	C23—C24	1.499 (3)
N4—C16	1.338 (3)	C24—C25	1.385 (3)
N4—C17	1.344 (3)	C24—C29	1.405 (3)
C1—C5	1.383 (3)	C25—C26	1.374 (3)
C1—C6	1.492 (3)	C25—H25	0.9300
C2—C3	1.372 (3)	C26—C27	1.391 (3)
C2—H2	0.9300	C26—H26	0.9300
C3—C4	1.371 (3)	C27—C28	1.373 (3)
C3—H3	0.9300	C27—H27	0.9300
C4—C5	1.381 (3)	C28—C29	1.408 (3)
C4—H4	0.9300	C29—C30	1.489 (3)
C5—H5	0.9300	C30—C31	1.494 (3)
C6—C10	1.387 (3)	C31—C32	1.389 (3)
C7—C8	1.376 (3)	C32—C33	1.373 (3)
C7—H7	0.9300	C32—H32	0.9300
C8—C9	1.371 (4)	C33—C34	1.388 (3)
C8—H8	0.9300	C33—H33	0.9300
C9—C10	1.378 (3)	C34—H34	0.9300
C9—H9	0.9300		
			
N3—Cd1—N1	166.26 (6)	N3—C11—C16	116.78 (18)
N3—Cd1—N2	114.20 (6)	C15—C11—C16	122.14 (19)
N1—Cd1—N2	71.82 (6)	N3—C12—C13	123.1 (2)
N3—Cd1—O4^i^	100.05 (6)	N3—C12—H12	118.4
N1—Cd1—O4^i^	92.77 (6)	C13—C12—H12	118.4
N2—Cd1—O4^i^	84.02 (6)	C12—C13—C14	118.1 (2)
N3—Cd1—N4	71.49 (6)	C12—C13—H13	120.9
N1—Cd1—N4	105.00 (6)	C14—C13—H13	120.9
N2—Cd1—N4	168.49 (6)	C13—C14—C15	119.7 (2)
O4^i^—Cd1—N4	85.11 (6)	C13—C14—H14	120.1
N3—Cd1—O2	85.54 (6)	C15—C14—H14	120.1
N1—Cd1—O2	81.21 (6)	C14—C15—C11	119.1 (2)
N2—Cd1—O2	99.17 (6)	C14—C15—H15	120.4
O4^i^—Cd1—O2	171.85 (6)	C11—C15—H15	120.4
N4—Cd1—O2	91.13 (6)	N4—C16—C20	121.5 (2)
O1—S1—O3	113.39 (12)	N4—C16—C11	117.42 (18)
O1—S1—O2	113.56 (10)	C20—C16—C11	121.1 (2)
O3—S1—O2	111.31 (11)	N4—C17—C18	123.2 (2)
O1—S1—C21	106.45 (10)	N4—C17—H17	118.4
O3—S1—C21	103.90 (10)	C18—C17—H17	118.4
O2—S1—C21	107.45 (9)	C19—C18—C17	118.7 (2)
O5—S2—O6	113.97 (11)	C19—C18—H18	120.7
O5—S2—O4	113.30 (10)	C17—C18—H18	120.7
O6—S2—O4	111.62 (12)	C18—C19—C20	119.0 (2)
O5—S2—C28	108.28 (10)	C18—C19—H19	120.5
O6—S2—C28	104.48 (10)	C20—C19—H19	120.5
O4—S2—C28	104.26 (9)	C19—C20—C16	119.6 (2)
S1—O2—Cd1	148.54 (9)	C19—C20—H20	120.2
S2—O4—Cd1^ii^	122.01 (10)	C16—C20—H20	120.2
C2—N1—C1	118.40 (18)	C34—C21—C22	119.58 (19)
C2—N1—Cd1	123.63 (14)	C34—C21—S1	114.81 (16)
C1—N1—Cd1	117.25 (13)	C22—C21—S1	125.42 (16)
C6—N2—C7	118.44 (18)	C31—C22—C21	118.18 (19)
C6—N2—Cd1	116.16 (13)	C31—C22—C23	117.20 (18)
C7—N2—Cd1	125.05 (14)	C21—C22—C23	124.61 (18)
C12—N3—C11	118.79 (18)	O7—C23—C22	122.5 (2)
C12—N3—Cd1	121.94 (14)	O7—C23—C24	119.4 (2)
C11—N3—Cd1	119.26 (14)	C22—C23—C24	118.09 (17)
C16—N4—C17	118.06 (19)	C25—C24—C29	120.49 (19)
C16—N4—Cd1	115.03 (14)	C25—C24—C23	117.80 (18)
C17—N4—Cd1	126.90 (15)	C29—C24—C23	121.70 (19)
N1—C1—C5	121.1 (2)	C26—C25—C24	120.4 (2)
N1—C1—C6	117.06 (18)	C26—C25—H25	119.8
C5—C1—C6	121.76 (19)	C24—C25—H25	119.8
N1—C2—C3	123.4 (2)	C25—C26—C27	119.4 (2)
N1—C2—H2	118.3	C25—C26—H26	120.3
C3—C2—H2	118.3	C27—C26—H26	120.3
C4—C3—C2	118.3 (2)	C28—C27—C26	121.3 (2)
C4—C3—H3	120.9	C28—C27—H27	119.4
C2—C3—H3	120.9	C26—C27—H27	119.4
C3—C4—C5	119.2 (2)	C27—C28—C29	119.72 (19)
C3—C4—H4	120.4	C27—C28—S2	116.19 (16)
C5—C4—H4	120.4	C29—C28—S2	123.97 (16)
C4—C5—C1	119.6 (2)	C24—C29—C28	118.45 (19)
C4—C5—H5	120.2	C24—C29—C30	116.71 (18)
C1—C5—H5	120.2	C28—C29—C30	124.78 (18)
N2—C6—C10	121.4 (2)	O8—C30—C29	122.7 (2)
N2—C6—C1	117.14 (17)	O8—C30—C31	119.8 (2)
C10—C6—C1	121.50 (19)	C29—C30—C31	117.46 (17)
N2—C7—C8	122.9 (2)	C32—C31—C22	121.0 (2)
N2—C7—H7	118.6	C32—C31—C30	117.17 (18)
C8—C7—H7	118.6	C22—C31—C30	121.86 (19)
C9—C8—C7	118.7 (2)	C33—C32—C31	120.3 (2)
C9—C8—H8	120.7	C33—C32—H32	119.9
C7—C8—H8	120.7	C31—C32—H32	119.9
C8—C9—C10	119.2 (2)	C32—C33—C34	119.2 (2)
C8—C9—H9	120.4	C32—C33—H33	120.4
C10—C9—H9	120.4	C34—C33—H33	120.4
C9—C10—C6	119.4 (2)	C21—C34—C33	121.7 (2)
C9—C10—H10	120.3	C21—C34—H34	119.1
C6—C10—H10	120.3	C33—C34—H34	119.1
N3—C11—C15	121.1 (2)		
			
O1—S1—O2—Cd1	−133.06 (19)	Cd1—N3—C11—C16	0.5 (2)
O3—S1—O2—Cd1	97.5 (2)	C11—N3—C12—C13	−1.3 (3)
C21—S1—O2—Cd1	−15.6 (2)	Cd1—N3—C12—C13	−179.92 (18)
N3—Cd1—O2—S1	135.7 (2)	N3—C12—C13—C14	0.3 (4)
N1—Cd1—O2—S1	−47.93 (19)	C12—C13—C14—C15	1.0 (4)
N2—Cd1—O2—S1	21.9 (2)	C13—C14—C15—C11	−1.3 (4)
O4^i^—Cd1—O2—S1	−90.6 (4)	N3—C11—C15—C14	0.3 (3)
N4—Cd1—O2—S1	−152.9 (2)	C16—C11—C15—C14	179.4 (2)
O5—S2—O4—Cd1^ii^	−153.24 (9)	C17—N4—C16—C20	−1.6 (3)
O6—S2—O4—Cd1^ii^	−22.97 (14)	Cd1—N4—C16—C20	179.43 (18)
C28—S2—O4—Cd1^ii^	89.25 (12)	C17—N4—C16—C11	177.09 (19)
N3—Cd1—N1—C2	−54.1 (3)	Cd1—N4—C16—C11	−1.9 (2)
N2—Cd1—N1—C2	−172.37 (19)	N3—C11—C16—N4	1.0 (3)
O4^i^—Cd1—N1—C2	104.84 (18)	C15—C11—C16—N4	−178.1 (2)
N4—Cd1—N1—C2	19.17 (19)	N3—C11—C16—C20	179.7 (2)
O2—Cd1—N1—C2	−69.64 (17)	C15—C11—C16—C20	0.6 (3)
N3—Cd1—N1—C1	116.1 (3)	C16—N4—C17—C18	0.8 (3)
N2—Cd1—N1—C1	−2.19 (14)	Cd1—N4—C17—C18	179.62 (17)
O4^i^—Cd1—N1—C1	−84.98 (15)	N4—C17—C18—C19	0.6 (4)
N4—Cd1—N1—C1	−170.65 (14)	C17—C18—C19—C20	−1.2 (4)
O2—Cd1—N1—C1	100.54 (15)	C18—C19—C20—C16	0.4 (4)
N3—Cd1—N2—C6	−169.45 (14)	N4—C16—C20—C19	1.1 (4)
N1—Cd1—N2—C6	−2.71 (14)	C11—C16—C20—C19	−177.6 (2)
O4^i^—Cd1—N2—C6	92.20 (15)	O1—S1—C21—C34	−125.08 (19)
N4—Cd1—N2—C6	73.0 (3)	O3—S1—C21—C34	−5.1 (2)
O2—Cd1—N2—C6	−80.24 (15)	O2—S1—C21—C34	112.94 (18)
N3—Cd1—N2—C7	17.4 (2)	O1—S1—C21—C22	49.9 (2)
N1—Cd1—N2—C7	−175.85 (19)	O3—S1—C21—C22	169.90 (19)
O4^i^—Cd1—N2—C7	−80.94 (18)	O2—S1—C21—C22	−72.0 (2)
N4—Cd1—N2—C7	−100.1 (3)	C34—C21—C22—C31	0.2 (3)
O2—Cd1—N2—C7	106.62 (18)	S1—C21—C22—C31	−174.63 (16)
N1—Cd1—N3—C12	−105.1 (3)	C34—C21—C22—C23	−178.8 (2)
N2—Cd1—N3—C12	8.35 (19)	S1—C21—C22—C23	6.4 (3)
O4^i^—Cd1—N3—C12	96.27 (17)	C31—C22—C23—O7	−160.9 (2)
N4—Cd1—N3—C12	177.60 (19)	C21—C22—C23—O7	18.1 (4)
O2—Cd1—N3—C12	−89.71 (17)	C31—C22—C23—C24	20.5 (3)
N1—Cd1—N3—C11	76.2 (3)	C21—C22—C23—C24	−160.5 (2)
N2—Cd1—N3—C11	−170.32 (15)	O7—C23—C24—C25	−14.3 (3)
O4^i^—Cd1—N3—C11	−82.40 (16)	C22—C23—C24—C25	164.3 (2)
N4—Cd1—N3—C11	−1.07 (15)	O7—C23—C24—C29	166.8 (2)
O2—Cd1—N3—C11	91.62 (16)	C22—C23—C24—C29	−14.5 (3)
N3—Cd1—N4—C16	1.55 (14)	C29—C24—C25—C26	4.1 (3)
N1—Cd1—N4—C16	−164.57 (14)	C23—C24—C25—C26	−174.7 (2)
N2—Cd1—N4—C16	123.0 (3)	C24—C25—C26—C27	−0.1 (4)
O4^i^—Cd1—N4—C16	103.87 (15)	C25—C26—C27—C28	−3.0 (4)
O2—Cd1—N4—C16	−83.37 (15)	C26—C27—C28—C29	2.0 (3)
N3—Cd1—N4—C17	−177.3 (2)	C26—C27—C28—S2	178.08 (18)
N1—Cd1—N4—C17	16.59 (19)	O5—S2—C28—C27	108.37 (18)
N2—Cd1—N4—C17	−55.8 (4)	O6—S2—C28—C27	−13.4 (2)
O4^i^—Cd1—N4—C17	−74.98 (19)	O4—S2—C28—C27	−130.72 (17)
O2—Cd1—N4—C17	97.79 (19)	O5—S2—C28—C29	−75.71 (19)
C2—N1—C1—C5	−0.3 (3)	O6—S2—C28—C29	162.48 (18)
Cd1—N1—C1—C5	−171.01 (16)	O4—S2—C28—C29	45.2 (2)
C2—N1—C1—C6	177.15 (19)	C25—C24—C29—C28	−5.1 (3)
Cd1—N1—C1—C6	6.4 (2)	C23—C24—C29—C28	173.74 (19)
C1—N1—C2—C3	−0.1 (3)	C25—C24—C29—C30	172.3 (2)
Cd1—N1—C2—C3	169.98 (18)	C23—C24—C29—C30	−8.9 (3)
N1—C2—C3—C4	0.2 (4)	C27—C28—C29—C24	2.0 (3)
C2—C3—C4—C5	0.1 (4)	S2—C28—C29—C24	−173.74 (15)
C3—C4—C5—C1	−0.5 (4)	C27—C28—C29—C30	−175.1 (2)
N1—C1—C5—C4	0.6 (3)	S2—C28—C29—C30	9.1 (3)
C6—C1—C5—C4	−176.8 (2)	C24—C29—C30—O8	−153.2 (2)
C7—N2—C6—C10	0.2 (3)	C28—C29—C30—O8	24.0 (3)
Cd1—N2—C6—C10	−173.46 (16)	C24—C29—C30—C31	25.7 (3)
C7—N2—C6—C1	−179.50 (19)	C28—C29—C30—C31	−157.10 (19)
Cd1—N2—C6—C1	6.9 (2)	C21—C22—C31—C32	−1.9 (3)
N1—C1—C6—N2	−9.0 (3)	C23—C22—C31—C32	177.2 (2)
C5—C1—C6—N2	168.4 (2)	C21—C22—C31—C30	177.53 (19)
N1—C1—C6—C10	171.4 (2)	C23—C22—C31—C30	−3.4 (3)
C5—C1—C6—C10	−11.2 (3)	O8—C30—C31—C32	−21.7 (3)
C6—N2—C7—C8	1.2 (3)	C29—C30—C31—C32	159.4 (2)
Cd1—N2—C7—C8	174.19 (18)	O8—C30—C31—C22	158.9 (2)
N2—C7—C8—C9	−1.9 (4)	C29—C30—C31—C22	−20.1 (3)
C7—C8—C9—C10	1.2 (4)	C22—C31—C32—C33	2.2 (4)
C8—C9—C10—C6	0.1 (4)	C30—C31—C32—C33	−177.2 (2)
N2—C6—C10—C9	−0.8 (3)	C31—C32—C33—C34	−0.8 (4)
C1—C6—C10—C9	178.8 (2)	C22—C21—C34—C33	1.2 (4)
C12—N3—C11—C15	0.9 (3)	S1—C21—C34—C33	176.5 (2)
Cd1—N3—C11—C15	179.65 (16)	C32—C33—C34—C21	−0.9 (4)
C12—N3—C11—C16	−178.19 (19)		

Symmetry codes: (i) *x*, *y*−1, *z*; (ii) *x*, *y*+1, *z*.

### Hydrogen-bond geometry (Å, °)

**Table:**

*D*—H···*A*	*D*—H	H···*A*	*D*···*A*	*D*—H···*A*
C4—H4···O5^iii^	0.93	2.48	3.200 (3)	135
C5—H5···O4^iii^	0.93	2.53	3.239 (3)	133
C14—H14···O2^iv^	0.93	2.54	3.267 (3)	136

Symmetry codes: (iii) −*x*+1, −*y*+1, −*z*+2; (iv) −*x*+2, −*y*, −*z*+1.
